# History of Pregnancy Loss Increases the Risk of Mental Health Problems in Subsequent Pregnancies but Not in the Postpartum

**DOI:** 10.1371/journal.pone.0095038

**Published:** 2014-04-14

**Authors:** Catherine Chojenta, Sheree Harris, Nicole Reilly, Peta Forder, Marie-Paule Austin, Deborah Loxton

**Affiliations:** 1 Research Centre for Gender, Health and Ageing, University of Newcastle, Newcastle, NSW, Australia; 2 Perinatal and Women's Mental Health Unit, St John of God Health Care and University of New South Wales, Sydney, NSW, Australia,; Harvard Medical School, United States of America

## Abstract

While grief, emotional distress and other mental health conditions have been associated with pregnancy loss, less is known about the mental health impact of these events during subsequent pregnancies and births. This paper examined the impact of any type of pregnancy loss on mental health in a subsequent pregnancy and postpartum. Data were obtained from a sub-sample (N = 584) of the 1973-78 cohort of the Australian Longitudinal Study on Women's Health, a prospective cohort study that has been collecting data since 1996. Pregnancy loss was defined as miscarriage, termination due to medical reasons, ectopic pregnancy and stillbirth. Mental health outcomes included depression, anxiety, stress or distress, sadness or low mood, excessive worry, lack of enjoyment, and feelings of guilt. Demographic factors and mental health history were controlled for in the analysis. Women with a previous pregnancy loss were more likely to experience sadness or low mood (AOR = 1.75, 95% CI: 1.11 to 2.76, p = 0.0162), and excessive worry (AOR = 2.01, 95% CI: 1.24 to 3.24, p = 0.0043) during a subsequent pregnancy, but not during the postpartum phase following a subsequent birth. These results indicate that while women who have experienced a pregnancy loss are a more vulnerable population during a subsequent pregnancy, these deficits are not evident in the postpartum.

## Introduction

Pregnancy loss including medical termination, miscarriage, ectopic pregnancy and stillbirth are commonly associated with grief following the event [1]. Additionally, women who have experienced a pregnancy loss are more likely to experience an adverse mental health problem such as depression and anxiety following the loss [2]–[7]. Less is known about the mental health outcomes during subsequent pregnancies and following healthy births; however, results from limited studies indicate that women with adverse reproductive and pregnancy histories were more likely to experience psychological distress in a subsequent pregnancy [8], [9] and postnatally [10]. While previous studies have examined subsequent mental health during pregnancy or during the postspartum, no studies to date have examined the mental health outcomes during both the pregnancy and the postpartum for the same women who have experienced prior loss.

This study examines the impact of any type of pregnancy loss on mental health in subsequent pregnancies and postpartum using a large-scale broadly representative sample of Australian women. It is expected that women who have experienced a previous loss will be at greater risk of a range of poor psychological outcomes in subsequent pregnancies and postpartum after other known risk factors have been taken into account such as previous mental health issues and demographic factors.

## Methods

### Participants

**Figure 1:**
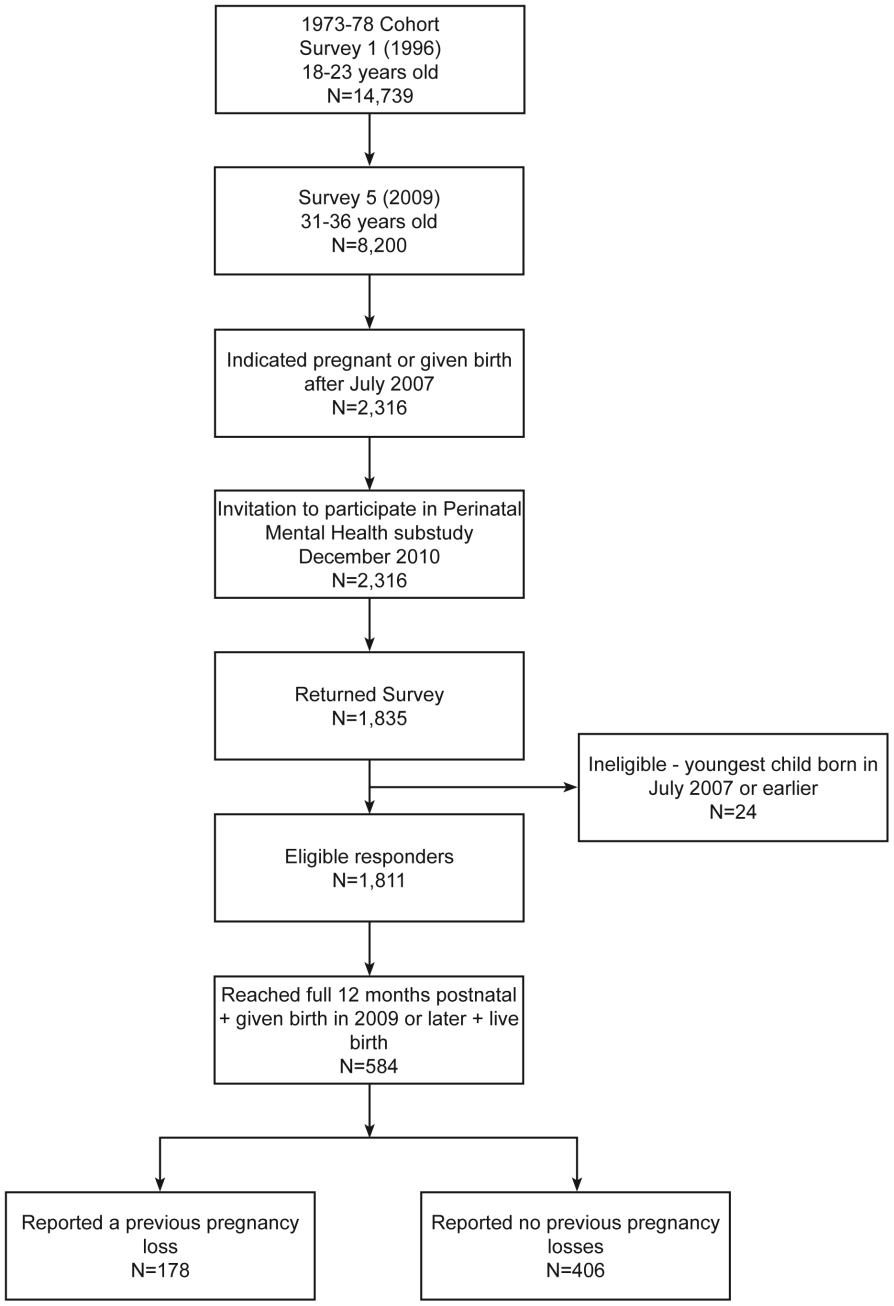
Recruitment and sample selection for the Perinatal Mental Health substudy within the Australian Longitudinal Study of Women's Health.

Participants for this study were from the Australian Longitudinal Study on Women's Health (ALSWH). ALSWH is a prospective cohort study which has examined the health of over 40,000 Australian women in three age cohorts; 1973-78, 1946-51 and 1921-26 since 1996. Participants were recruited via the Medicare (health insurance) database, and complete mailed surveys on a three-yearly basis. Further information has been published elsewhere [11], [12]. Questions in the ALSWH are based on the biopsychosocial view of health. The following analysis examines data collected from the 1973-78 cohort (N = 14,217) who were aged 18–23 years in 1996 and have completed up to six surveys to date. The ALSWH Perinatal Mental Health (PNMH) substudy was conducted in 2011 and was restricted to women who had responded to ALSWH Survey 5 in 2009 and who were currently pregnant or had recently given birth at the time of survey. Primiparous women who were unsure of their pregnancy status were excluded from the sampling frame. A total of 2316 eligible participants were invited to participate in the PNMH substudy, with 1835 surveys returned (ie a 79% response rate). There were 24 respondents who did not meet eligibility criteria and were subsequently excluded from the substudy. Details of the sample selection can be found in Figure 1.

Data were used from both the PMNH substudy (2011) as well as the surveys from the main ALSWH study. In order to evaluate outcomes for the full postnatal period of 12 months, only women who had reached 12 months since birth and who had given birth to their youngest child in 2009 or later and whose youngest child was born live were included for the analysis. Additionally two women who had not completed the items on adverse reproductive and pregnancy events in Survey 5 were excluded. The resulting sample size for the analysis was N = 584.

Approvals for the project were granted by the Human Research Ethics Committees of the University of Newcastle (HREC Ref: H-2010-0031; March 2010), the University of Queensland (HREC Ref: 20100000411; April 2010) and the University of New South Wales (HREC Ref: 10412; November 2010). Written informed consent was obtained from all participants for the collection of sub-study data, and to linkage of sub-study data with previously collected Main Survey data. To preserve anonymity and confidentiality, data were de-identified prior to analysis. ALSWH data is routinely archived in the Australian Data Archive (www.ada.edu.au).

### Outcome measures

#### Emotional issues during pregnancy and during the postnatal period

Respondents were asked if they had experienced a range of emotional issues during the first half of pregnancy and/or the second half of pregnancy such as depression, anxiety, stress or distress, sadness or low mood, lack of enjoyment or interest in things, feelings of guilt or excessive worry. Each of these items were single item questions and were then combined over the pregnancy period to create a dichotomous variable during pregnancy (yes/no).

Similarly, respondents were asked if they had experienced the same range of emotional issues after the birth of their youngest child (0–3 months after the birth, and/or 4–12 months after). These items were collapsed to create a dichotomous variable for self-reported emotional issues during the postnatal period (yes/no).

### Explanatory variables

#### Adverse reproductive and pregnancy events

Participants were asked if they had ever experienced a termination due to medical reasons, miscarriage, ectopic pregnancy or stillbirth at any time prior to the birth of their youngest child. These items were collapsed to create a dichotomous variable for adverse reproductive and pregnancy events, with a response of ‘yes’ to any of the single items coded as ‘yes’ to experiencing any type of adverse reproductive or pregnancy event prior to the birth of their youngest child. This was the main explanatory variable of interest.

#### Mental health history

Information was drawn from the ALSWH Main Surveys for Young Women [Survey 2 (S2; in 2000), Survey 3 (S3; in 2003), Survey 4 (S4; in 2004), Survey 5 (S5, in 2009, if completed prior to the index pregnancy)]. Women were asked: (a) “In the last three years have you been diagnosed or treated for” (S3, S4, S5) or “In the last four years/more than four years ago (S2) have you been told by a doctor that you have: i) postnatal depression; ii) depression (not postnatal); iii) anxiety disorder; iv) other major mental illness” (S4 and S5 only), with yes/no response options for each condition. (b) if they had experienced i) antenatal depression; ii) postnatal depression; iii) antenatal anxiety; iv) postnatal anxiety, with yes/no response options for each condition, for each previous pregnancy/birth (S5 only). (c) “In the last 12 months, have you had any of the following i) depression; ii) episodes of intense anxiety; iii) other mental health problems” (S4 and S5 only), with response options of ‘never’, ‘rarely’, sometimes' or ‘often’ for each condition.

A woman was considered to have a history of depression, anxiety or other mental health issue if she gave an affirmative response to any of the conditions listed in (a) or (b), and/or if she responded “often” to any of the conditions listed in (c).

#### Partner status

Respondents were asked about their current marital status in the PNMH sub study survey. Responses of ‘Never married’, ‘Separated’, ‘Divorced’, and ‘Widowed’ were classified as ‘Single’. Responses of ‘Married’, ‘De facto (opposite sex), and ‘De facto (same sex)’ were classified as ‘Partnered’.

#### Language background

Respondents were asked if they usually spoke a language other than English at home in Survey 1. Responses of ‘No, I speak only English at home’ were classified as an English speaking background (ESB). All other responses were classified as a Non-English speaking background (NESB).

#### Area of residence

Respondents were classified as living in major city, regional or remote from their postcode given at the time of Survey 5 based on the ARIA Plus classification of remoteness [13].

#### Parity

Respondents were classified as having no other children, 1 other child, 2 or more other children from data collected from Survey 5 and the PNMH study.

#### Income management

Respondents were asked how they manage on their available income. Responses of ‘It is impossible’, ‘It is difficult all the time’ and ‘It is difficult some of the time’ were classified as ‘Difficult’. Responses of ‘It is not too bad’ and ‘It is easy’ were classified as ‘Not difficult’.

#### Education

Respondents were asked to indicate their highest level of education completed. Responses were collapsed into four categories; ‘No formal qualifications/Year 10 or equivalent’, ‘Year 12’; ‘Trade/apprenticeship/certificate/diploma’; and ‘University degree/Higher university degree’.

#### Employment status

Respondents were asked to indicate their employment status at the time of the birth of their youngest child. Responses were collapsed to create a dichotomous variable of ‘employed’ versus ‘not employed’.

### Statistical Methods

Logistic regression models were used to assess associations between emotional issues and past adverse reproductive and pregnancy events, with each emotional issue experienced during pregnancy and during the postnatal period being analysed as a separate outcome variable. Initially univariate logistic regressions were analysed to identify any significant or marginally significant relationships (using p<.10 as the inclusion criterion). Multivariate models which adjusted for demographic variables and previous mental health issues were then analysed. All logistic regressions, using the logit link function, were performed in SAS 9.2 [14]. Post-hoc power analysis revealed the power to detect an absolute difference between 5% and 10% ranged from 7–86%, depending on the chosen outcome. The advantage of rich, longitudinal data from a broadly representative cohort of Australian women means that meaningful conclusions can still be made from the results.

## Results

General characteristics of the study population are reported in Table 1. Of the 584 participants in the study, 178 (30.5%) had reported experiencing a pregnancy loss prior to the birth of their youngest child. The most commonly reported adverse reproductive or pregnancy event was miscarriage (n = 162, 27.7%). The occurrence of termination for medical reasons (n = 16, 2.7%), stillbirth (n = 12, 2.1%) and ectopic pregnancy (n = 8, 1.4%) were reported with much lower frequency (see Table 2).

**Table 1:** Demographic characteristics (N = 584).

	n (%)
**Mental health history:**	
Yes	57 (9.8%)
No	527 (90.2%)
**Residential area:**	
Major city	340 (58.2%)
Regional	225 (38.5%)
Remote	17 (2.9%)
*Missing*	*2 (0.3%)*
**Highest educational level:**	
Year 10/No formal qualifications	26 (4.5%)
Year 12	57 (9.8%)
Trade/Certificate/Diploma	133 (22.8%)
University degree or higher degree	366 (62.7%)
*Missing*	*2 (0.3%)*
**Income management stress:**	
Difficult	234 (40.1%)
Not difficult	348 (59.6%)
*Missing*	*2 (0.3%)*
Parity:	
No other children	156 (26.7%)
One other child	277 (47.4%)
Two or more other children	151 (25.9%)
**Partner status:**	
Partnered	566 (96.9%)
Unpartnered	17 (2.9%)
*Missing*	*1 (0.2%)*
Language Background:	
English speaking background	547 (93.7%)
Non-English speaking background	32 (5.5%)
*Missing*	*5 (0.9%)*
**Employment status:**	
Employed	423 (72.4%)
Unemployed	157 (26.9%)
*Missing*	*4 (0.7%)*

**Table 2:** Prevalence of adverse reproductive and pregnancy events.

	N (%)
**Any adverse event**	**178 (30.5%)**
Stillbirth	12 (2.1%)
Miscarriage	162 (27.7%)
Termination (medical reasons)	16 (2.7%)
Ectopic pregnancy	8 (1.4%)

Nearly half of the women (45.5%) indicated that they experienced at least one emotional issue during their most recent pregnancy with anxiety being the most commonly reported emotional issue (26.4%), followed by stress or distress (25.0%) and sadness or low mood (20.2%). During the postnatal period just over half the women (51.9%) reported at least one emotional issue during the postnatal period with sadness and low mood being the most commonly reported emotional issue (30.1%), followed by stress or distress (25.3%) and anxiety (22.3%).Table 3 presents the prevalence for all the emotional issues reported during pregnancy and the postnatal period.

**Table 3:** Prevalence of emotional issues during pregnancy and the postnatal period.

	During pregnancy	During postnatal period
	n (%)	n (%)
**Any emotional issue**	**266 (45.5%)**	**303 (51.9%)**
Depression	52 (8.9%)	73 (12.5%)
Anxiety	154 (26.4%)	130 (22.3%)
Stress or distress	146 (25.0%)	148 (25.3%)
Sadness or low mood	118 (20.2%)	176 (30.1%)
Lack of enjoyment or interest in things	62 (10.6%)	85 (14.6%)
Feelings of guilt	62 (10.6%)	107 (18.3%)
Excessive worry	105 (18.0%)	80 (13.7%)

After controlling for demographic variables and previous mental health issues, having a previous pregnancy loss was significantly associated with higher rates of some emotional issues during the most recent pregnancy (Table 4). After adjusting for demographic factors and previous mental health issues, the odds of reporting excessive worry during pregnancy were more than double for women who had experienced a pregnancy loss (OR = 2.01, 95% CI: 1.24 to 3.24, p = 0.0043). Women who had experienced a pregnancy loss were also more likely to report sadness or low mood (OR = 1.75, 95% CI: 1.11 to 2.76, p = 0.0162). There was no evidence to suggest a previous pregnancy loss was associated with depression, anxiety, stress or distress, feelings of guilt or with lack of enjoyment or interest in things during pregnancy.

**Table 4:** Associations between previous pregnancy loss and emotional issues in a subsequent pregnancy.

		Unadjusted		Adjusted^1^	
Outcome	Adverse event	OR [95% CI]	pvalue	OR [95% CI]	pvalue
**Depression**	No	1	-	-	-
	Yes	1.35 [0.75, 2.45]	0.32	-	-
**Anxiety**	No	1	-	-	-
	Yes	1.33 [0.90, 1.97]	0.15	-	-
**Stress or distress**	No	1	-	-	-
	Yes	1.31 [0.88, 1.96]	0.18	-	-
**Sadness or low mood**	No	1	-	-	-
	Yes	1.69 [1.11, 2.58]	0.0141	1.75 [1.11, 2.76]	0.0162
**Lack of enjoyment or interest in things**	No	1	-	-	-
	Yes	1.10 [0.62, 1.93]	0.75	-	-
**Feelings of guilt**	No	1	-	-	-
	Yes	1.10 [0.62, 1.93]	0.75	-	-
**Excessive worry**	No	1	-	1	-
	Yes	1.68 [1.08, 2.61]	0.0201	2.01 [1.24, 3.24]	0.0043

1 Adjusted for demographic variables and previous mental health issue as shown in Table 1

There was no evidence to suggest an association between prior pregnancy loss and any emotional issue during the postnatal period (see Table 5).

**Table 5:** Results of logistic regression analyses for emotional issues during the postnatal period.

		Unadjusted		Adjusted^1^	
Outcome	Adverse event	OR [95% CI]	pvalue	OR [95% CI]	pvalue
**Depression**	No	1	-	-	-
	Yes	0.91 [0.53, 1.56]	0.73	-	-
**Anxiety**	No	1	-	-	-
	Yes	1.07 [0.70, 1.62]	0.77	-	-
**Stress or distress**	No	1	-	-	-
	Yes	1.23 [0.82, 1.83]	0.31	-	-
**Sadness or low mood**	No	1	-	-	-
	Yes	0.94 [0.64, 1.38]	0.75	-	-
**Lack of enjoyment or interest in things**	No	1	-	-	-
	Yes	0.82 [0.49, 1.38]	0.46	-	-
**Feelings of guilt**	No	1	-	-	-
	Yes	1.08 [0.69, 1.69]	0.75	-	-
**Excessive worry**	No	1	-	-	-
	Yes	1.35 [0.83, 2.22]	0.23	-	-

1 Adjusted for demographic variables and previous mental health issue as shown in Table 1.

## Discussion

This study has been the first to demonstrate that women who have experienced a prior pregnancy loss were more likely to experience sadness or low mood and excessive worry during a subsequent pregnancy when compared to women with no history of pregnancy loss, even after controlling for previous mental health issues and demographic factors. In the postpartum, these emotional issues were not associated with previous pregnancy loss. Importantly, this study reported these findings for the same group of women, unlike past research which has focussed on either the pregnancy or postpartum period, indicating that while a range of mental health issues may be common in a pregnancy following a pregnancy loss, these mental health issues are not more likely to occur for these women in the postpartum following a live birth.

These findings are consistent with previous research that indicated an adverse mental health impact of previous adverse reproductive and pregnancy events in a subsequent pregnancy [8], [9] but are inconsistent with findings for the postpartum [10]. However, the current study was the first to examine this impact in a longitudinal context and to examine both pregnancy and the postpartum period following a live birth. The use of a non-clinical sample additionally helps to demonstrate that women who have experienced a prior loss are not only at greater risk of diagnosable conditions such as depression and anxiety, but also a wide range of other mental health issues such as stress, sadness and excessive worry. Clinically this is important information as it points to the need to monitor these women more closely during subsequent pregnancies and if necessary, to offer them anxiety or stress management interventions at this time. Such interventions will potentially benefit the woman herself, but may also reduce adverse obstetric and neonatal [15] and longer term offspring outcomes [16], which might be adversely impacted by poor mental health in pregnancy.

The limitations of the current study should be acknowledged. Firstly, while the individual types of pregnancy losses are not comparable due to small numbers of some events, the overall number of participants with pregnancy losses are adequate for meaningful analyses to be conducted. Secondly, it was not possible to measure the time between the adverse pregnancy and reproductive event and the birth of the child in the current study and this has been shown to be a significant factor in previous literature [9]. Nonetheless our results show that regardless of timing of pregnancies, women who have experienced a prior pregnancy loss are at greater risk of experiencing mental health issues in a subsequent pregnancy. Additionally, caution should be taken when generalising the results of the current study. While the ALSWH sample is broadly representative of Australian population, the PNMH subsample consists of mothers who are older than average, therefore the results may not apply to women outside of these age ranges. However, it must also be noted that in Australia, this age range incorporates the time when most women are having children.

Our findings indicate that women who have experienced a loss should not only be targeted for mental health interventions immediately following the loss of their pregnancy, but also in any subsequent pregnancy. While enquiry about pregnancy loss in the last 12 months is recommended in standard psychosocial risk screening in pregnancy in the *Clinical Practice Guidelines for Depression and Related Disorders - Anxiety, Bipolar Disorder and Puerperal Psychosis - in the Perinatal Period* [17], [18], the current findings indicate the importance of inquiring about any pregnancy loss, regardless of timing. Such inquiries permit the need for appropriate interventions to be explored with expectant mothers, which can both improve their experience of pregnancy as well as help to protect the mother and infant from stress related perinatal complications.
